# Automatic real-time occupational posture evaluation and select corresponding ergonomic assessments

**DOI:** 10.1038/s41598-022-05812-9

**Published:** 2022-02-08

**Authors:** Po-Chieh Lin, Yu-Jung Chen, Wei-Shin Chen, Yun-Ju Lee

**Affiliations:** Department of Industrial Engineering and Engineering Management (R924), College of Engineering, National Tsing Hua University, No. 101, Sec. 2, Kuang-Fu Rd., Hsinchu City, 30013 Taiwan

**Keywords:** Engineering, Biomedical engineering

## Abstract

The objective is to develop a system to automatically select the corresponding assessment scales and calculate the score of the risk based on the joint angle information obtained from the imaged process (OpenPose) via image-based motion capture technology. Current occupational assessments, for example, REBA, RULA, and OWAS were used to evaluate the risk of musculoskeletal disorders. However, the assessment result would not be reported immediately. Introducing real-time occupational assessments in different working environments will be helpful for occupational injury prevention. In this study, the decision tree was developed to select the most appropriate assessment method according to the joint angles derived by OpenPose image process. Fifteen operation videos were tested and these videos can be classified into six types including maintenance, handling, assembly, cleaning, office work, and driving. The selected ergonomic assessment method by our developed decision tree in each condition are consistent with the recommendation of the Labour Research Institute. Moreover, the high-risk posture could be identified immediately and provide to the inspector for further evaluation on this posture rather than the whole operation period. This approach provides a quick inspection of the operation movements to prevent musculoskeletal injuries and enhances the application of the scale assessment method in different industrial environments.

## Introduction

The occupational injuries of the worker are defined as danger or harm connected with the working environment. The cause of occupational injury may be physical, chemical, biological, human, or others factors^1^. Due to novel technological advances, many devices or techniques were developed to prevent or detect the possible risk of occupational injury. However, there are still many operations in the industry that rely on the execution of the traditional workforce, which have more potential risk of causing occupational injuries. The most common occupational injury type is musculoskeletal, and the main factors include repeatability task, overload, or working postures^2^. It has been reported from a decade ago studies that musculoskeletal disorders are strongly associated with the physical and social environmen^3^. According to the other report^1^, about 65.16% of the workers with physically uncomfortable during the previous year. The musculoskeletal pain was the most symptom, especially occurred in the shoulder (41.31%), neck (32.25%), lower back, or waist (31.03%). Therefore, the musculoskeletal injuries are still the most common injury among all the occupational diseases until now^1,4,5^. Musculoskeletal injuries, in some cases are permanently irreversible, would reduce the working efficiency of the workers, and lead to high cost and time-consuming medical treatment or rehabilitation. Hence, how to prevent musculoskeletal injuries is become more and more important^6^ and many studies focus on the estimation of the risk of the work-related musculoskeletal disorder in the workplace, the musculoskeletal posture, working duration, weight-bearing, or other factors^7^.

The occupational assessment methods are commonly used to estimate the risk of the occupational injuries^8–10^. The investigator would scoring the degree of the risk by observing the working posture, loading, the environment based, or the other factors according to the guideline of each assessment method^8^. Because the occupational assessment is convenient and can be simply utilized in the industry, it is the most commonly used evaluation method such as the Ovako Working Posture Analyzing System (OWAS)^11^, Rapid Entire Body Assessment (REBA)^12^, and Rapid Upper Limb Assessment (RULA)^13^. The assessment method would be selected according to the characteristic of the operational movement^10^ and the risk of the posture would be underestimated if the inappropriate assessment method is selected. Therefore, some studies focus on the comparison of the results between two different assessment methods or conduct two assessments at the same time^10,14^. It is important to choose the proper assessment, therefore, one of our objectives is to propose a strategy for selecting the appropriate assessment method.

Moreover, these ergonomic assessment methods are performed manually by the expert based on the identification of postures, actions, or joint angles^15^ which would cause subjective bias^16^, and the high-risk posture during an operational period would be ignored. The conventional method is difficult to provide the improvement immediately or long-term monitoring of the risk of occupational injury because the occupational assessment needs to be conducted offline. In general, the average number of frames in each video is 500 (around 18 s), and it is time-consuming and easy to make mistakes to review these frames manually. Therefore, the automation of picking high-risk frames out for further investigation would be a valuable and practical methodology.

Until now, real-time risk assessment for musculoskeletal disorder (MSD) has been a challenging research problem^17^. In order to tackle this issue, many studies proposed a variety of techniques such as attaching sensors on the human body, optical motion capture system, and image-based motion analysis. For example, the Ergonomic Assessment Work-Sheet (EAWS) and RULA assessment methods have been automatically evaluated by the information acquired from the IMU sensor^15,18^. But this technique would affect the subjects’ motion and causing the inaccurate evaluation results. The optical method is to capture the location in space of the reflective markers which are stuck on the specific joints or position of the human body, and then derive the joint angle or posture of the human body^19,20^. However, it is hard to set up the optical motion capture system in the workplace and the accuracy of the locating the reflective marker would be affected by the different environment. As long as the acquisition method needs a sensor or optical marker attached to the human body, it will affect the movement of the operation and the assessment results. The image-based technique is to calculate the motion and joint angles by analyzing the RGB image without stick markers or sensors on human body. The body posture and motion video of the subject could be recorded during long time operation, and the general camera can be an appropriate tool that is easy to set up in the factory to collect the information. The OpenPose Model is an RGB image-based posture recognition model by using deep learning^21^, which is developed by Carnegie Mellon University. The skeleton of the body can be implemented, and the joint angle can be calculated by the joint position. Some studies published recently also proposed the vision-based method (OpenPose) to localize body joints and recognize the human pose^22–25^. The outputs of OpenPose were the 2D coordinates of 18 key points. RGB images can be collected easily in the working place. It provides the opportunity to obtain the joint and activity information in real-time by using OpenPose image process without any attached sensor on the human body. Although, in some case of body or object occlusion, the key point derived from OpenPose will lose and causing inaccurate calculation of joint angle^26^. RULA and REBA assessment were also automatically estimated based on the joint angles by using deep learning methods^17,27–29^. These studies suggest that the estimated joint angles based on OpenPose might be reliable. Therefore, this study aims to adopt this image processing technology to calculate the joint angle and then integrating it with the decision tree developed in this study to select the most appropriate assessment method from commonly used methods in the industrial for the evaluated operation movement. The commonly used assessment methods include REBA, OWAS, and RULA, which cover the action of the entire body, repeat operation, and specific upper limbs.

## Materials and methods

Firstly, the accuracy for joint angle calculation from OpenPose was verified by the validation experiment in this study. Secondly, the joint angles were calculated by the joint position derived from OpenPose. Subsequently, a decision tree was developed to automatically select the corresponding assessment method and calculate the score based on the joint angle via the selected method. The general assessment methods such as REBA, OWAS, and RULA, were included in the decision tree due to their applicability and suitability. Finally, the highest score frame which means the high-risk posture could be identified subsequently. The approach developed by this study provides an automated and comprehensive approach to evaluate the risk and the prevent the musculoskeletal injuries.

### Validation experiments

**Figure 1:**
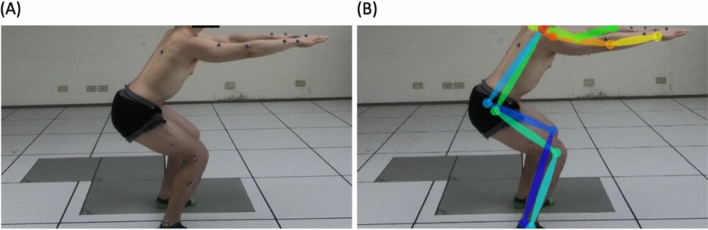
(**A**) The reflective markers on the body (**B**) OpenPose implanted human skeleton model.

**Figure 2:**
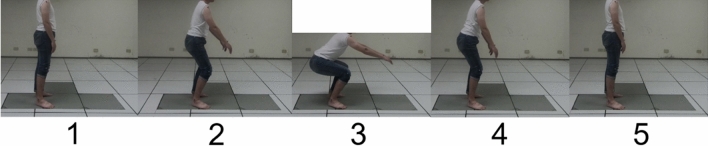
Five key frames in the squat task.

The joint angles calculated from the position information which are derived from the OpenPose were validated by the optical camera (Vicon, Oxford, UK), which served as the golden standard in various studies concerning the accuracy of positioning. The absolute errors of joint angle from each joint were calculated to evaluate whether the system is acceptable for this application. The reflective markers were attached to the bony landmarks of the subject and the position information was acquired by the VICON optical camera. While the image camera (GoPro HERO 6 Black) was set up at 3 m away from the right side of the subject acquiring the imaging information required by OpenPose (Fig. 1). The subjects were free from any musculoskeletal disorder and neurologic disease that could affect the performance of the movement. The experiment was approved by the National Tsing-Hua University Institutional Review Board (REC No.: 10710HE070) and compliance with relevant guidelines and regulations, and all subjects provided written informed consent before taking part in the experimental process. The subjects were instructed to perform the squat task during the experiment. Start with feet slightly wider than hip-width apart, keep the chest up and shift the weight onto the heels as pushing hips into a sitting position. And then lower the hips until thighs are almost parallel to the floor and go back up to the starting position. Five joint angles were calculated, including neck, trunk, knee, shoulder, and elbow and the selected five keyframes for joint angle validation within the squat task (see Fig. 2). Finally, the mean absolute errors of joint angle for each keyframes represent the applicability of OpenPose, which we discuss further in the results and discussion section.

### System illustration

**Figure 3:**
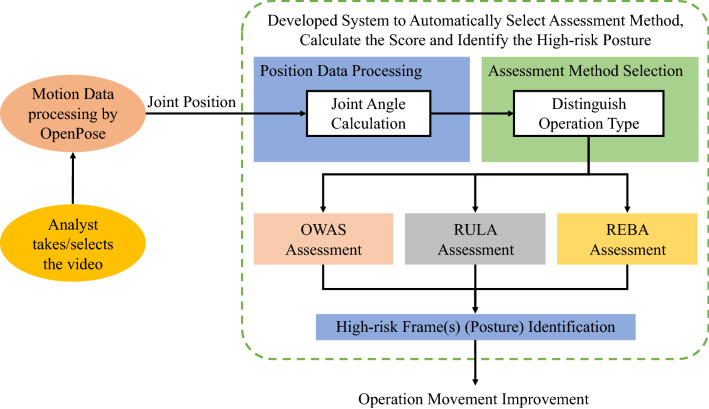
The overall scheme of evaluation module based on image processing technology.

Figure 3 shows the flow chart of the developed evaluation system which has four steps and will be described in the following sections. The four steps including Video Selection and Motion Data Processing, 2D Joint Angles Computation, Scale Selection, and Score Calculation and Evaluation.

#### Video selection and motion data processing

**Figure 4:**
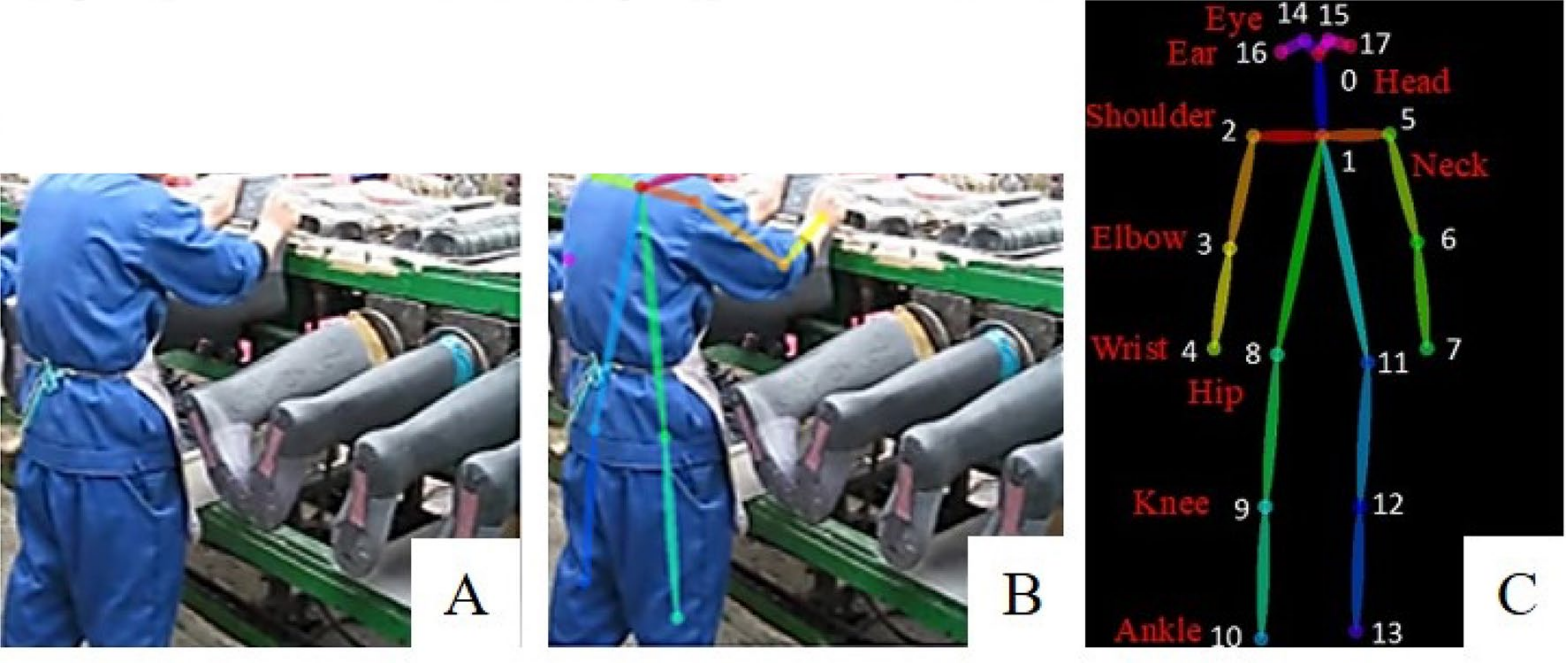
(**A**) Screenshots of actions in assembly type operations (3)^31^. (**B**) The OpenPose implanted human skeleton model. (**C**) The 18 key-points output from OpenPose.

According to the general working types proposed by the Labour Research Institute^30^, there are six types of operating activities such as computer users, machine maintenance, manual handling, assembly, clearing, and driving. Fifteen videos with at least 30 frames per second, including these six types of operation, were selected from online resources (YouTube, LLC). Each video was taken the whole body and trimmed into one operational cycle from the start posture to the same posture without repetitive movement. Subsequently, the operational cycle videos were taken as inputs to OpenPose, which is the open-source real-time system for the human 2D pose detection approach^22^. Take the manual handling operations as an example, the keyframe of the captured image is shown in Fig. 4A and the embedded human skeleton information by OpenPose is shown in Fig. 4B. The outputs of OpenPose were the 2D coordinates of 18 key points in 30 frames per second. An example of OpenPose human skeleton is shown in Fig. 4C, including the head, neck, bilateral eyes, ears, hips, knees, ankles, shoulders, elbows, and wrists.

#### 2D joint angles computation

These positions of each skeleton point were projected onto the parallel plane to the camera imaging and the joint angle was calculated by the vector of each limb and its adjacent limb. We employed the inverse trigonometric function (Formular (1)) to calculate the angle between two vectors, each vector consists of the position of the joint and its adjacent point.

1 $$ {\text{A}}_{{\text{i}}} = {\text{a}}\cos \left( {\frac{{{\text{v}}_{{\text{j}}} \cdot {\text{v}}_{{\text{k}}} }}{{\left| {{\text{v}}_{{\text{j}}} } \right|\left| {{\text{v}}_{{\text{k}}} } \right|}}} \right) \times \frac{180}{\uppi } $$

The three relevant points were selected to define the required posture or joints (Fig. 4C). For the head posture, the positions of ear, neck, and hip were selected. For the trunk segment, the positions were neck, hip, and knee. For the knee joint, the positions were hip, knee, and ankle. For the shoulder joint, the positions were hip, shoulder, and elbow. For the elbow joint, the positions were shoulder, elbow, and wrist. However, the current OpenPose skeleton did not include the positions of hand for the wrist joint computation. The wrist joint angle was set as zero in the current study.

#### Scale selection

**Figure 5:**
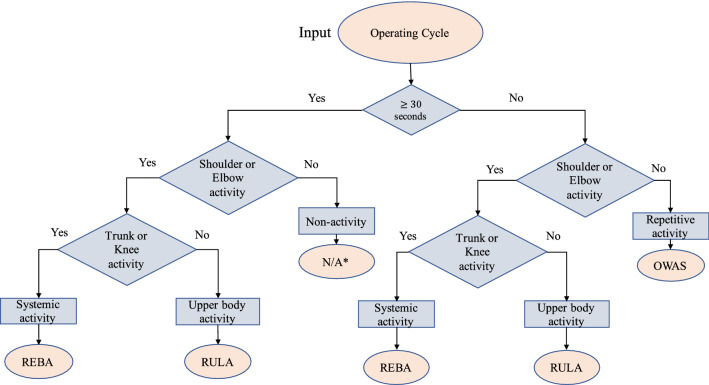
The flow chart of the assessment method selection. *REBA* Rapid entire body assessment, *OWAS* Ovako working posture analysing system, *RULA* Rapid upper limb assessment; *Not Applicable: suggest select other method rather than these three.

The third step was to select the appropriate assessment method by the decision tree developed in this study, which distinguish the posture characteristics of each operational movement. The assessment method selection flowchart is shown in Fig. 5. The three decision points including (1) whether the operation was a repetitive activity, (2) whether the operation was upper limb activity, and (3) whether the operation was lower limb activity. The criterion for repetitive activities was the time of the operational cycle less than 30 s, which means less than 900 frames of the output from OpenPose. The criteria for upper and lower limb activity referred to the action level of the shoulder, elbow, trunk, and knee defined in REBA, for example, the upper limb activity was defined as the joint angle of shoulder or elbow covers all action levels. Similarly, the lower limb activity was defined as the trunk or knee position angle covers all action levels. According to the characteristic of each assessment method recognized by the Labor Research Institute, the methods, OWAS, RULA, and REBA, are applicable for repetitive work, upper extremity work activity, and systemic activity respectively. Finally, four decision options were REBA for whole body (upper and lower) activity in both repetitive and non-repetitive operation, OWAS for upper and repetitive activity, RULA for upper activity in both repetitive and non-repetitive operation, and “Not Applicable” indicated the operation was not applicable in these methods and suggested adopting other method.

#### Score calculation and evaluation

After the assessment method selection, each joint and posture were scored according to the selected method (OWAS, RULA, or REBA). The risk score was summed of the total score for each frame and then the posture of the frames with the highest risk score would be identified and provide to the ergonomists for further evaluation and improvement in the future.

## Results

Table 1 shows the error of each joint angle between the VICON system and the OpenPose, the mean absolute error is 8.01 ± 1.37 for the knee, 3.23 ± 1.15 for the shoulder, and 4.24 ± 1.66 for the elbow.

**Table 1:** The error of each joint angle in squat task.

Keyframes	Neck	Trunk	Knee	Shoulder	Elbow
1	6.05	2.73	9.05	4.31	7.44
2	3.96	2.17	7.95	2.28	3.80
3	4.68	2.09	9.86	2.33	3.20
4	3.41	3.22	5.95	2.31	2.74
5	3.68	3.62	7.24	4.94	4.01
Mean ± SD	4.36 ± 0.95	2.77 ± 0.59	8.01 ± 1.37	3.23 ± 1.15	4.24 ± 1.66

Table 2 shows the result of the three activity decision nodes, including “repetitive activity”, “upper limb (shoulder or elbow) activity”, and “lower limb (trunk or knee) activity” for fifteen operational videos. The assessment method suggested by the decision tree are shown in Table 2, which are corresponding to the recommend assessment by the Institute of Labor, Occupational Safety and Health in Taiwan.

**Table 2:** The result of decision node and assessment of each operation.

Task		Decision tree			Assessment result	Recommend assessment (Institute of Labor, Occupational Safety and Health in Taiwan)
#	Type	Repetitive	Upper limb	Lower limb		
1	Assembly^32^	Y	Y	Y	REBA	OWAS, RULA, REBA
2	Assembly^31^	Y	N	N	OWAS	OWAS, RULA, REBA
3	Assembly^31^	Y	Y	Y	REBA	OWAS, RULA, REBA
4	Maintenance^33^	Y	N	N	OWAS	OWAS, RULA, REBA
5	Maintenance^34^	Y	Y	N	RULA	OWAS, RULA, REBA
6	Maintenance^35^	Y	N	N	OWAS	OWAS, RULA, REBA
7	Computer users^36^	Y	Y	N	RULA	OWAS, RULA, REBA
8	Computer users^36^	Y	N	N	OWAS	OWAS, RULA, REBA
9	Manual handling^37^	Y	Y	N	RULA	RULA, REBA
10	Manual handling^38^	Y	Y	Y	REBA	RULA, REBA
11	Manual handling^39^	Y	Y	Y	REBA	RULA, REBA
12	Cleaning^32^	N	Y	N	RULA	OWAS, RULA, REBA
13	Cleaning^40^	N	Y	Y	REBA	OWAS, RULA, REBA
14	Cleaning^40^	Y	Y	Y	REBA	OWAS, RULA, REBA
15	Driving^41^	Y	Y	N	RULA	RULA

**Figure 6:**
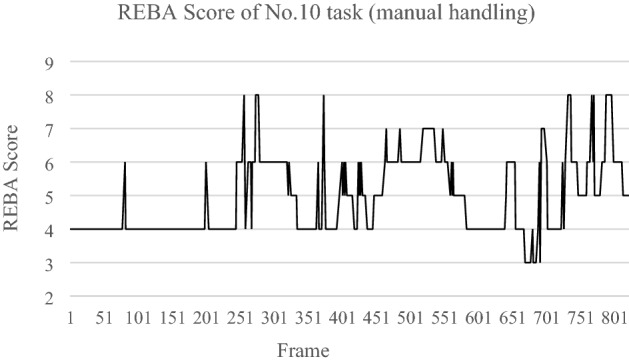
The total score in REBA through the selected working period.

Taking one of manual handling operation (video No. 10) as an example, the risk scores of each joint such as neck, legs, trunk posture, lower arm, upper arm, and wrist. REBA assessment method was selected by the decision tree to evaluate this video. In the REBA assessment, four subscores indicate different evaluations such as score A for neck, trunk, and leg analysis; score B for arm and wrist analysis; score C is combined by score A and B; activity score evaluates the movement ability of the worker. The values variate starts from the first frame for these three subscores, therefore the changes in the REBA score occurred during the working period. Figure 6 shows the entire REBA score distribution and it is easy to identify that the highest score is 8 in video No10. According to the instruction of the assessment method^12^, the score 8 to 10 would be classified as “High Risk”.

**Figure 7:**
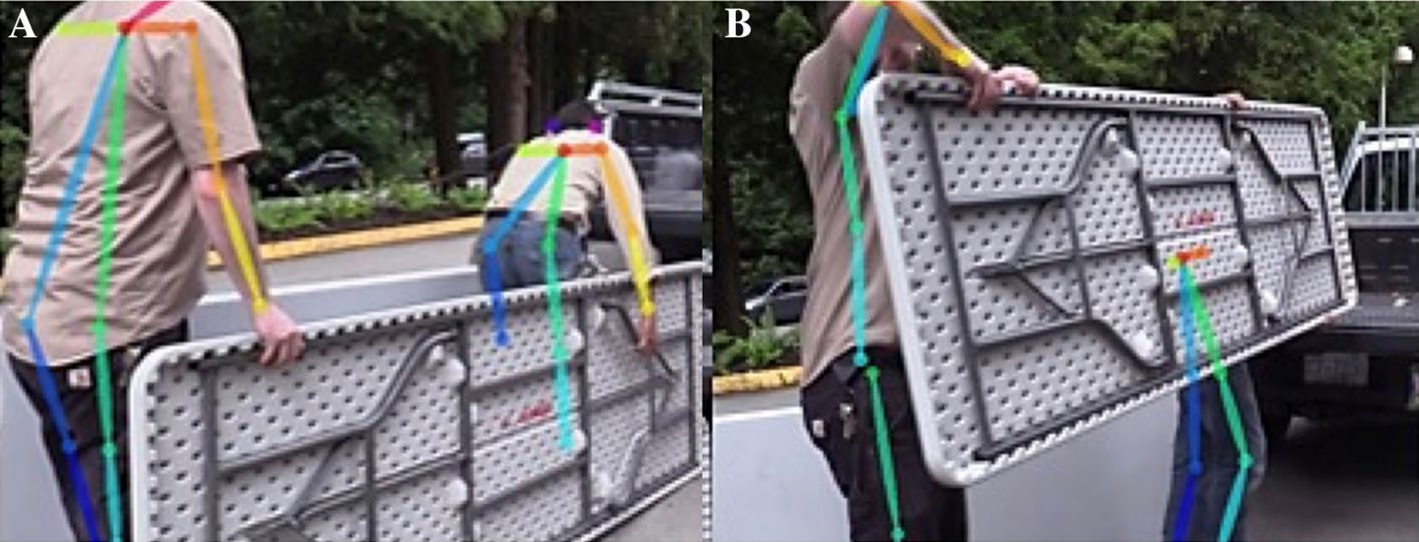
Pictures are with the images with the skeleton model (**A**) Frame 272 of video 10 and (**B**) Frame 370 of video 10.

The amount of the high-risk frames could be counted as shown in Fig. 6, from 250 to 400th frame, and 700th to 800th frame, and Table 3 shows that there were 27 frames (3.3% of total frames) with the highest risk (score 8) during the working period. The image and the skeleton model of the selected key frames 272nd and 370th frames are in Fig. 7.

**Table 3:** The maximal score and level of MSD risk of total operation.

#	Task type	Assessment result	Total frame	Maximal score	Level of MSD risk	# of frame with high risk (%)
1	Assembly^32^	REBA	646	7	Medium	0 (0%)
2	Assembly^31^	OWAS	319	2	Low	0 (0%)
3	Assembly^31^	REBA	862	7	Medium	0 (0%)
4	Maintenance^33^	OWAS	430	2	Low	0 (0%)
5	Maintenance^34^	RULA	178	5	Medium	0 (0%)
6	Maintenance^35^	OWAS	414	2	Low	0 (0%)
7	Computer users^36^	RULA	178	5	Medium	0 (0%)
8	Computer users^36^	OWAS	415	2	Low	0 (0%)
9	Manual handling^37^	RULA	788	6	Medium	0 (0%)
10	Manual handling^38^	REBA	822	8	High	27 (3.3%)
11	Manual handling^39^	REBA	366	6	Medium	0 (0%)
12	Cleaning^32^	RULA	997	6	Medium	0 (0%)
13	Cleaning^40^	REBA	925	9	High	713 (77.1%)
14	Cleaning^40^	REBA	864	8	High	152 (17.6%)
15	Driving^41^	RULA	388	6	Medium	0 (0%)

Frames with high MSD risk (420)/Total frames of 15 operations (8592) = 10.4%.

Table 3 shows the maximal score and highest risk level of 15 operations. There are three tasks that were classified as high risk (No. 10, 13, and 14), and eight tasks were classified as medium risk (No. 1, 3, 5, 7, 9, 11, 12, and 15). The percentage of the high-risk frame was 3.3%, 77.1%, and 17.6% of each high-risk operation. The system developed in this study identified there are 420 frames (10.4%) with high-risk level from total frames (8592) from 15 videos. The postures of these frames during the working period should be provided to the ergonomic expert for further investigation.

## Discussion

The OpenPose has been widely used to recognize the dynamic posture of humans in the videos^24,25,42–44^. The result of the validation experiment in this study shows that the errors are less than 8.01 degrees on average of each joint (the maximum error is knee joint which is 9.86 degree in one of the tests) between OpenPose and VICON system. These differences are acceptable for the assessment methods used in this study and provide tolerances to the developed system such as the placement of the camera doesn’t need to be precise, some systematic errors of joint angle calculation, and the different definition of joint center locations^21,45^. Furthermore, the assessment scores derived from the developed system using the joint angle information acquired by both OpenPose and VICON are the same. Hence the deviation of the system and OpenPose would not cause inaccurate results and acceptable for the automatic assessment of operational movement.

The results of assessment method selection and high-risk posture identification are corresponding to the conventional practice. The fifteen videos were successfully assigned to the proper assessment method, which consistent to the recommendation by the Institute of Labor, Occupational Safety and Health. The frames of the high-risk posture recognized by the developed system are the same as the assessment from the ergonomic expert, which were around 10.4% (Table 3) of the working period. Therefore, the system is able to objectively quantify the joint angle in each movement and is a time-saving tool on identifying the postures that need to be improved which costs enormous time in traditional expert investigation.

The automatic ergonomic assessment system has been proposed by not only using IMU sensors for the EAWS scale^15^ and the RULA^18^ but also the vision-based approach for RULA and REBA^17,27,46^. However, these studies had decided the specific ergonomic assessment method prior to input the information into the automatic assessment system. The present study includes three ergonomic assessment methods instead of a specific one. The selected videos cover as many operation types as possible to validate the developed decision tree more broadly. Nevertheless, the potential incorrect decision might happen when the operation action is complicated or too many limb occlusions. Further study should focus on more variety of actions to increase the applicability of the decision tree. The decision tree based on four joint angles (shoulder, elbow, trunk, and knee) was used to check whether the operation with or without the “repetitive activity,” “upper limb activity,” and “lower limb activity.” By including four joint angles, the assessment would be selected specifically and precisely based on the principle of the assessment method. Therefore, the decision tree developed in this study is applicable for whole-body assessment and able to assign the evaluated movement to the most appropriate method such as OWAS, RULA, or REBA.

Subsequently, the conventional ergonomic assessment is performed manually by the expert based on the identification of postures and actions^15,16^, which means every single frame of the operation movement video needs to be investigated individually to find out the risky posture. The whole evaluation process is time-consuming, moreover, the potential risk posture might be ignored due to the subjective effects by interrater. The developed system, however, could automatically calculate all the scores for each frame, and especially the frame with the highest score will be identified for further investigation and improvement. According to the result, three operations with high MSD risk were identified from 15 operations (Table 3) for imminently examination, and eight operations as the medium risk. The result of subsequent evaluation shows that the No.10 task (manual handling) with a score of 8 in 27 frames among 822 frames (3.3%) (Fig. 6). Two of the high-scoring frames shown in Fig. 7 indicate high-risk poses with a large joint angle in the waist (Fig. 7A) and elbow (Fig. 7B). With this information, an ergonomic expert can focus on 3.3% of the entire video which is the highest risk movement during the operation. In this way, the evaluation time for high-risk movement identification could be reduced by about 90%.

The advantage of vision-based method is that the whole-body activity and position could be simultaneously captured by a camera in working space without the cooperation of the subject or attaching sensors. However, some features of the operation could not be obtained by the video such as the reaction force and pressure on the floor. Although the weight estimation of an object from an image might be predicted by importing a vast database into the machine learning model^47^. Liu proposed video-based motion capture and force estimation frameworks for comprehensive ergonomic risk assessment, but the error of the estimated force attains 9.5 N (23.6%)^48^. Another study also proposed a model to estimate the workload on joints by combining the 3D posture data (estimated from 2D image) and foot pressure data^49^, however, the result shows that the error of estimated loads on key body joints is 15%. As the aforementioned restrictions, the limitation of this study is that we did not adopt the force features in our system, and the workload weight needs further investigation by the ergonomic expert. Furthermore, the present study demonstrated the application of conducting video frames and image keypoints identification (OpenPose) combined with the Decision Tree for ergonomic evaluations during working. Although the current approach achieved the reasonable selections, comparisons between the different machine learning approaches and confirmation from the ergonomic experts would need further investigation.

## Conclusions

The system was successfully developed to calculate joint angles via the key point matrix from OpenPose. Most importantly, the decision flow with three nodes could determine the corresponding ergonomic assessment method for the evaluation of occupational working pose. During the assessment process, the high-risk posture could be automatically identified, which saves time in evaluating the operational working period and does not omit certain high-risk posture that ignored by the human evaluation.
